# Genetic Variation in the Histamine Production, Response, and Degradation Pathway Is Associated with Histamine Pharmacodynamic Response in Children with Asthma

**DOI:** 10.3389/fphar.2016.00524

**Published:** 2017-01-04

**Authors:** Bridgette L. Jones, Catherine M. T. Sherwin, Xiaoxi Liu, Hongying Dai, Carrie A. Vyhlidal

**Affiliations:** ^1^Department of Pediatrics University of Missouri-Kansas City, Children's Mercy Hospitals and ClinicsKansas City, MO, USA; ^2^Division of Pediatric Pharmacology and Therapeutic Innovation, Children's Mercy Hospitals and ClinicsKansas City, MO, USA; ^3^Division of Allergy, Asthma and Clinical Immunology, Children's Mercy Hospitals and ClinicsKansas City, MO, USA; ^4^Children's Mercy Hospitals and ClinicsKansas City, MO, USA; ^5^Division of Clinical Pharmacology, Department of Pediatrics, University of Utah School of MedicineSalt Lake City, UT, USA; ^6^Department of Pharmacology and Toxicology, University of Utah College of PharmacySalt Lake City, UT, USA; ^7^University of Utah College of PharmacySalt Lake City, UT, USA; ^8^Division of Health Services and Outcomes Research, Children's Mercy Hospitals and ClinicsKansas City, MO, USA

**Keywords:** histamine, pharmacodynamics, pharmacogenetics, asthma, biomarker

## Abstract

**Introduction:** There is growing knowledge of the wide ranging effects of histamine throughout the body therefore it is important to better understand the effects of this amine in patients with asthma. We aimed to explore the association between histamine pharmacodynamic (PD) response and genetic variation in the histamine pathway in children with asthma.

**Methods:** Histamine Iontophoresis with Laser Doppler Monitoring (HILD) was performed in children with asthma and estimates for area under the effect curve (AUEC), maximal response over baseline (Emax), and time of Emax (Tmax) were calculated using non-compartmental analysis and non-linear mixed-effects model with a linked effect PK/PD model. DNA isolation and genotyping were performed among participants to detect known single nucleotide polymorphisms (SNPs) (*n* = 10) among genes (*HDC, HNMT, ABP1, HRH1, HRH4*) within the histamine pathway. General linear model was used to identify associations between histamine related genetic variants and measured histamine PD response parameters.

**Results:** Genotyping and HILD response profiles were completed for 163 children. *ABP1 47 C/T, ABP1 4107, and HNMT-1639 C/T*were associated with Emax (*ABP1* 47 CC genotype mean Emax 167.21 vs. CT/TT genotype mean Emax 139.20, *p* = 0.04; *ABP1* 4107 CC genotype mean Emax 141.72 vs. CG/GG genotype mean Emax 156.09, *p* = 0.005; *HNMT*-1639 CC genotype mean Emax 132.62 vs. CT/TT genotype mean Emax 155.3, *p* = 0.02). In a stratified analysis among African American children only, *ABP1* and *HNMT* SNPs were also associated with PD response; *HRH4* 413 CC genotype was associated with lower Emax, *p* = 0.009.

**Conclusions:** We show for the first time that histamine pathway genetic variation is associated with measureable changes in histamine response in children with asthma. The variability in histamine response and impact of histamine pathway genotype is important to further explore in patients with asthma so as to improve disease phenotyping leading to more personalized treatments.

## Introduction

Asthma is among the most common chronic diseases in children and adults. Despite considerable advances in the treatment of the disease, many patients continue to experience persistent symptoms. It is reported that up to 50% of children do not achieve adequate asthma control on top line therapies such as inhaled steroids (Szefler et al., 2002). It is important to better understand the inflammatory mediators involved in asthma pathogenesis so as to improve disease phenotyping and therapeutic outcomes. Histamine is an important mediator in the pathophysiology of asthma in children and adults. In addition to mediating symptoms of the allergic response (e.g., itching, sneezing, rhinitis), the amine exerts effects in the lung. Inhalation of histamine in the lung causes direct bronchoconstriction. Furthermore, activation of histamine receptors (H1, H2, H3, H4) throughout the body results in cytokine production, is involved in dendritic cell regulation, and modifies the Th1 and Th2 responses (Jones and Kearns, 2011). Antihistamines have been shown to be beneficial in reducing asthma exacerbations and even decreasing risk of asthma among those with specific asthma phenotypes (e.g., allergic asthma) (Grant et al., 1995; Warner, 2001). Understanding histamine pharmacodynamic (PD) response profiles in patients with asthma may provide an important surrogate endpoint for improving phenotyping asthma and for the evaluation of the effects of histamine in disease pathogenesis and treatment response.

Epicutaneous histamine testing (e.g., skin prick test) is the gold standard method for the evaluation of allergic disease in adults and children. The method is also used as a PD marker in the evaluation of antihistamine efficacy and duration of effect. PD histamine response may also be important in discriminating asthma phenotypes which may most significantly benefit from therapeutic agents that affect the allergic response pathway via interruption of the histamine response (e.g., antihistamines). We have previously reviewed the benefits of Histamine Iontophoresis with Laser Doppler monitoring (HILD) vs. the standard the epicutaneous skin prick test in discerning differences in histamine response between individuals. We observed that HILD provides a dynamic and robust characterization of histamine response (Jones et al., 2009). Additionally, among children with allergic rhinitis we observed that pharmacodynamic response to histamine varies among children and adults with allergic disease whereby children were able to be classified into distinct groups based on the magnitude of response profiles (Jones et al., 2013). Differences in the biological response to histamine may be relevant to disease pathogenesis and to therapeutic response to treatments that attenuate histamine action (e.g., antihistamines).

The histamine production, response, and degradation pathway includes: Histadine decarboxylase (HDC), responsible for production of histamine from histidine; H1-receptor (H1R), H2-receptor(H2R), H3-receptor (H3R), H4-receptor (H4R), receptors responsible for exerting histamine effects in the body; Histamine-N-methlytransferase (HNMT), responsible for degradation of ~80% of histamine in the body for elimination; and Diamine Oxidase (DAO), responsible for degradation of ~20% of histamine in the body for elimination(Jones and Kearns, 2011). Genetic variation within genes responsible for histamine production (*HDC*), response (*HRH1, HRH4*), and degradation (*HNMT, ABP1*) have been previously described (García-Martín et al., 2009). Single nucleotide polymorphisms (SNP) in these genes have been associated with asthma and allergic disease in children and adults of different racial and ethnic populations. We have previously observed that variants in *HRH1* and *HNMT* were associated with allergic asthma in children and specifically among African American children (Anvari et al., 2015). Although, several SNPs have been associated with allergic disease and asthma, the functional relevance of the majority of variants is unknown. The most thoroughly investigated SNP within the histamine pathway is *HNMT* 314 C/T. This SNP has been shown to result in alterations in protein folding of the enzyme and decreased enzymatic activity and has been associated with asthma and atopic dermatitis (Kennedy et al., 2008; Szczepankiewicz et al., 2010). Defects in histamine degradation may lead to accumulation of the amine and exaggerated and/or prolonged response to histamine at the receptor. Defects in histamine handling and/or alterations at the receptor may alter disease pathogenesis and antihistamine efficacy. Therefore, the aim of our study reported here was to explore genetic variation within the histamine pathway genes in relation to histamine pharmacodynamic response in children with asthma.

## Materials and methods

### Study population

All study participants were enrolled via convenience sampling in this cross-sectional study after obtaining parental permission and, when appropriate (i.e., age ≥ 7), child assent. This study was approved by the Children's Mercy Institutional Review Board (IRB# 10-10-192). Children with asthma were enrolled from Allergy, Asthma, and Immunology outpatient clinics at Children's Mercy in Kansas City, MO. Asthma was defined by ≥12% post-bronchodilator reversibility in forced expiratory volume in 1 s (FEV1) or by an Allergy/Asthma specialist diagnosis based on clinical symptoms in children unable to perform spirometry. All participants discontinued antihistamines (H1 receptor and H2 receptor inverse agonists) at least 10 days prior to the study and could not have used tricyclic antidepressants or systemic steroids 30 days prior to study participation.

### HILD

HILD was performed on the anterior surface of the forearm as previously described (Jones et al., 2009). A solid-state, single-frequency laser probe was inserted into the center of an iontophoresis chamber attached to a laser Doppler blood flow monitor (DRT4, Moor Instruments Ltd, Wilmington, Delaware) on the volar surface of the forearm. A second laser Doppler control probe was placed at a distance of at least 1 cm from the iontophoresis site as per previously described methods. Then, 200 μL of histamine dihydrochloride solution (1%) (Sigma Chemical Ltd, Dorset, UK), dissolved in a 2% methylcellulose gel (Sigma Chemical Co, St Louis, Missouri) was placed in the reservoir of the iontophoresis chamber. A platinum electrode in the iontophoresis chamber was connected to the positive terminal of a constant current source. For iontophoresis, constant anodal current (50 μA) was applied for 10 s. Values for small vessel blood flow at each of the probe sites were simultaneously calculated by software package provided by the manufacturer (Version2, Moor Instruments Ltd, Devon, UK) and given in perfusion units (flux). Baseline small vessel blood flow was assessed for 2 min before histamine iontophoresis. After histamine iontophoresis, blood flow was continuously assessed until blood flow measurements returned to baseline or for a total period up to 2 h.

Data collected from HILD monitoring was re-sampled using an averaging algorithm. The raw individual data (up to 3600 data points per subject) was first evenly divided into 200 segments with each containing approximately 18 observations. Within each segment, every data point was examined for potential outliers based on interquartile range rule. An averaged signal was calculated by taking the mean of signals within each segment. The corresponding time was assigned by taking the median time point within the segment. With this approach, each individual data set was reduced to 200 observations. A final screening was conducted manually to select 10–15 data points per subject to further reduce the size, while maintaining a realistic representation of the histamine response over time curve. The re-sampled data sets were then pooled together and subjected to pharmacometric modeling. NONMEM v7.3 (ICON Dev. Soln., Ellicott City, MD) was used to build the population PK/PD model. A population PK/PD linked effect model was developed to describe the histamine response over time monitored by HILD. Secondary modeling analysis provided parameter estimates for area under the effect curve (AUEC), and relative maximal response over baseline (Emax) and time of maximal response (Tmax) (Liu et al., 2016).

### DNA extraction and genotyping

Five milliliter of blood was collected into a glass tube containing ACD or calcium EDTA anticoagulant, mixed by repeated inversion and either stored for up to 7 days at 4°C or immediately frozen at −80°C. Genomic DNA was extracted from blood using the Illustra Blood Genomic Prep Mini Spin Kit (GE Healthcare, Piscataway, NJ). Genotyping assays were performed on genomic DNA (12–16 ng) using commercially available TaqMan assays to detect the following SNPs of interest: rs1049793 (*ABP1* 47 C/T), rs10156191 (*ABP1* 4107 C/G), rs1049742 (*ABP1* 995 C/T), rs17740607 (*HDC* 92 C/T), rs901865 (*HRH1*-17 C/T), rs11665084 (*HRH4* 413 C/T), rs6430764 (*HNMT*-1639 C/T), rs2071048 (*HNMT* 464 C/T), rs1050900 (*HNMT* 3′UTR A/T), and rs11558538 (*HNMT* 314 C/T) (Applied Biosystems, Foster City, CA) and KAPA Probe Fast qPCR master mix (Kapa Biosystems, Boston, MA) according to manufacturer's recommendations. SNPs were chosen based on their potential functional significance and previously investigated variants (see Supplementary Table 1), as well as those with an expected minor allele frequency (MAF) ≥2% within our expected participant population (Preuss et al., 1998; Sasaki et al., 2000; Hon et al., 2006; García-Martín et al., 2009). All samples were performed in duplicates to rule out random error.

### Statistical analysis

Concordance with Hardy-Weinberg equilibrium was confirmed for each SNP using available online software (Rodriguez, 2008). General linear model was used to compare genotype to pharmacodynamic parameters Emax, Tmax, and AUEC. Data were corrected for race and asthma type (allergic vs. non-allergic). Allergic categorization was defined by at least one positive skin prick test to a panel of common aeroallergens. In the dominant analysis, three genotypes were reclassified as wild type vs. non-wild type. In the stratified analysis, subjects were divided by race and analyzed within Caucasians and African Americans separately. Statistical significance was claimed with *p*-value < 0.05. Statistical analyses were performed using SAS 9.4 (Cary, NC).

## Results

*N* = 163 children were included in the final analysis of the study. Patient demographics are presented in Table 1.

**Table 1:** **Demographic characteristics of participants**.

**Characteristics**	**Mean ±*SD* or % (*N*)**
Age (years)	12.1 ± 3.1
Male	58 (94)
White (non-Hispanic)	54 (88)
Black (non-Hispanic)	35 (57)
Hispanic	11 (18)
Weight (kg)	52.1 ± 22.3
Height (cm)	150.6 ± 15.8
BMI	22.2 ± 6.8
Allergic Asthma	48 (78)

*Body Mass Index (BMI)*.

*Demographic characteristics expressed in Mean ± standard deviation (SD) or percentages (%) and number (N)*.

Emax and Tmax values were associated with genotype for *ABP1* and *HNMT* after correcting for race and asthma type. Genotype analysis revealed differences in Emax for *ABP1* 4107 C/G SNP (CC 141.72; CG 161.4; GG 142.06, *p* = 0.007). We also found an association between genotype and Tmax for *HNMT* 3′UTR A/T (AA 18.87, AT 18.48, TT 40.46, *p* = 0.001) (Supplementary Table 2).

**Figure 1:**
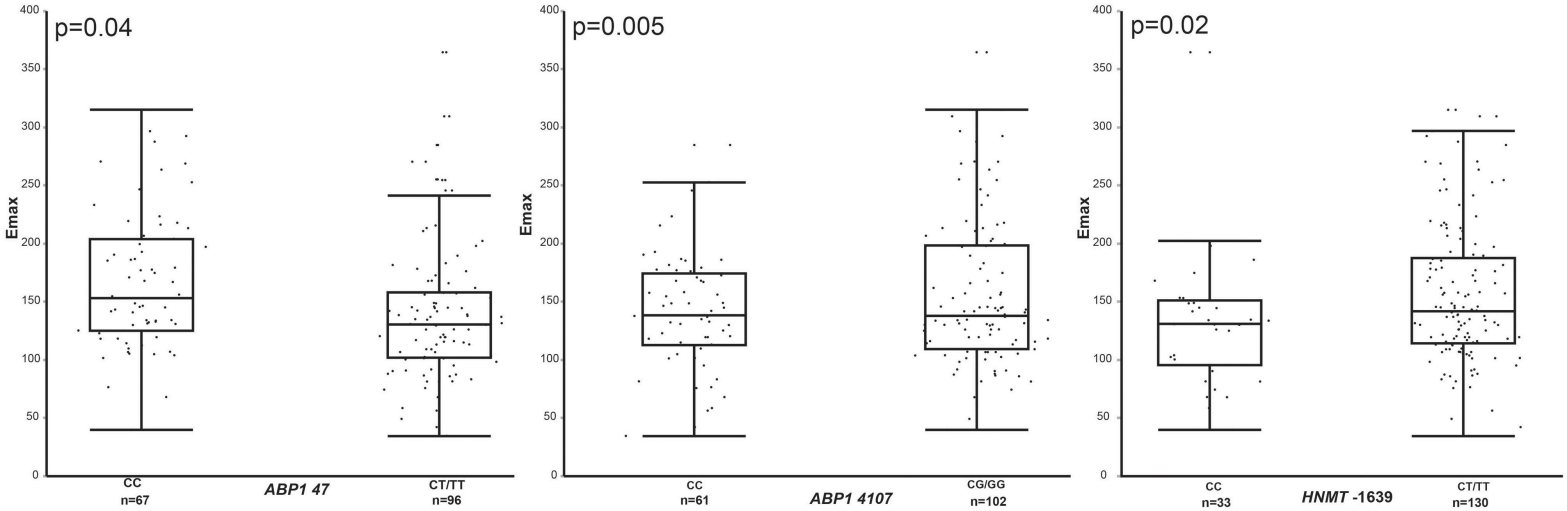
**Comparison of Emax values in wild-type homozygous vs. variant containing genotype children with asthma for ***ABP1*** 47, ***ABP1*** 4107, and ***HNMT***-1639**.

Dominant genotype analysis revealed an association of Emax with *ABP1* 47 C/T, *ABP1* 4107 C/G, and *HNMT*-1639 C/T SNPs (*ABP1* 47 C/T, CC 167.21 vs. CT/TT 139.20, *p* = 0.04; *ABP1* 4107 C/G, CC 141.72 vs. CG/GG 156.09, *p* = 0.005; *HNMT*-1639 C/T, CC 132.62 vs. CT/TT 155.3, *p* = 0.02) (Figure 1).

We did not observe an association between other investigated SNPs and pharmacodynamic response for (Supplementary Table 2).

**Figure 2:**
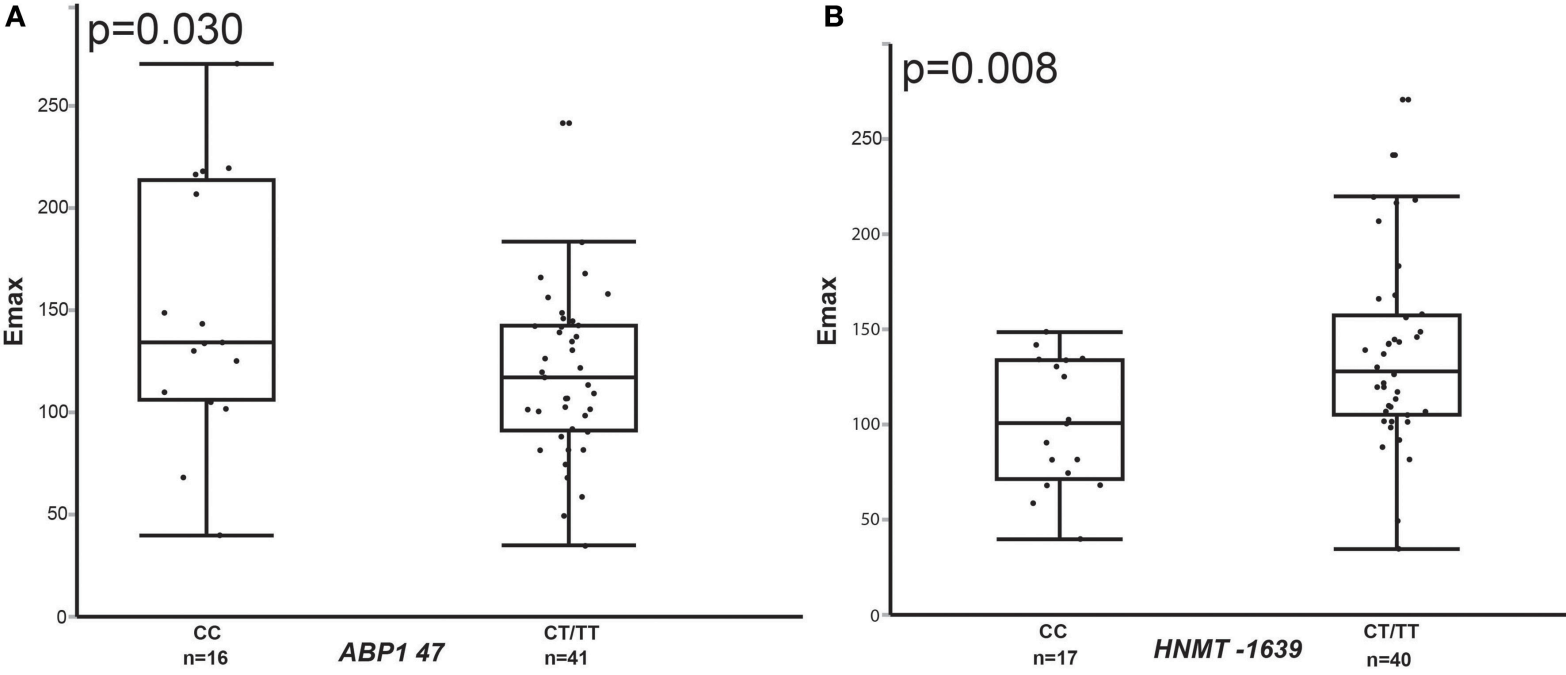
**Comparison of Emax values in wild-type homozygous vs. variant containing genotype African American children with asthma for ***ABP1*** 47 (A) and** ***HNMT*****-1639 (B)**.

**Figure 3:**
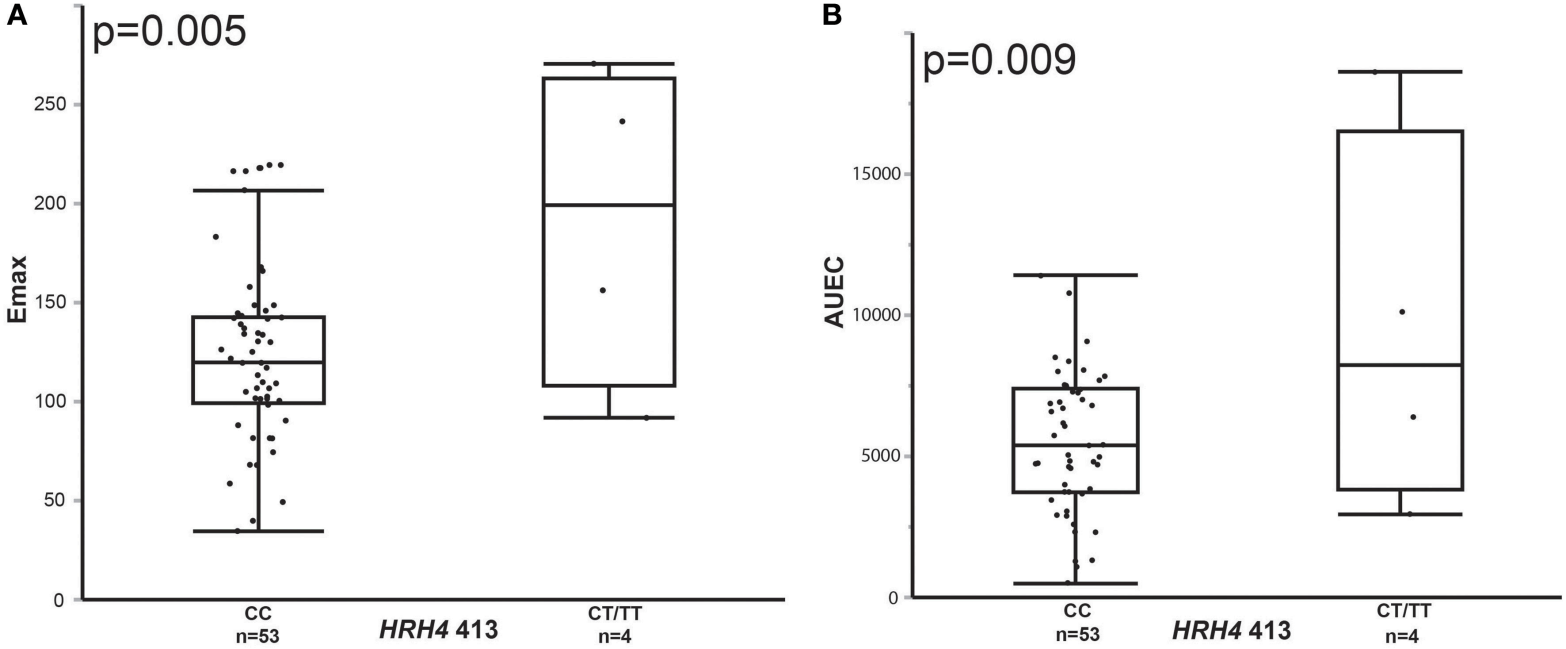
**Comparison of Emax (A) and AUEC (B) in wild-type homozygous vs. variant containing genotype African American children with asthma for ***HRH4 413*****.

We conducted further analysis of the relationship between genotype and pharmacodynamic response among stratified racial groups as asthma phenotype and/or pathophysiology may differ according to race. In a dominant genotype analysis among the stratified African American participants, after correction for asthma type, there was association between *ABP1* 47 C/T, *HRH4* 413 C/T, and *HNMT-*1639 C/T genotypes and pharmacodynamic response (Figures 2, 3). *ABP1* 47 CC genotype was associated with higher Emax (148.2 vs. 117.7 for CT/TT genotype, *p* = 0.030) (Figure 2A). *HNMT*-1639 CC genotype was associated with lower Emax (CC 100.85 vs. 137.06 CT/TT, *p* = 0.008) (Figure 2B). *HRH4* 413 CC genotype was associated with lower Emax (121.44 vs. 190.08 for CT/TT genotype, *p* = 0.005) as well as lower AUEC (CC 5525.04 vs. 9522.64 for CT/TT, *p* = 0.009) (Figures 3A,B respectively).

## Discussion

In this study we revealed an association between histamine pathway genetic variation and histamine PD response in children with asthma. These findings begin to elucidate the potential clinical impact of histamine genetic variation in children and adults with allergic and inflammatory disease. Furthermore, these results are important in further understanding how histamine genetic variation may affect asthma pathophysiology, severity, and therapeutic response.

Our study results suggest that genetic variation among genes responsible for histamine degradation (*HNMT* and *ABP1*) results in an increased histamine pharmacodynamic response profile in children with asthma. Previous investigations of the impact of histamine degrading enzyme genotype have revealed functional differences in enzyme kinetics and alterations in enzyme function. *ABP1* 4107 SNPs, which are associated with increased histamine response (increased Emax) in our study, are reported to result in missense mutations and decreased enzyme activity (Ayuso et al., 2007; Maintz et al., 2011). This previous *in-vitro* work lends biological plausibility to our findings in children with asthma. Defective histamine degradation would lead to accumulation of histamine in the body, saturation of the receptor site, and stabilization of the active form of the receptor, causing a more exaggerated response at the receptor site. These findings suggest that differences in genotype among the histamine pathway genes not only result in *in vitro* enzymatic changes but also result in measureable changes in histamine response in humans. We also found that *HNMT*-1639 genotype was associated with differences in Emax. Presence of the *HNMT*-1639 variant allele was associated with higher response to histamine. The *in vitro* functional consequence of the *HNMT*-1639 non-coding SNP is not currently known. As the SNP is present in the 5′-flanking region adjacent to the *HNMT* gene, it may affect translation and expression of the gene resulting in either increased or decreased expression or function of the enzyme and histamine degradation. Our current findings suggest that the variant may result in decreased expression/function of the enzyme leading to histamine accumulation and exaggerated response to histamine which we observed in our study. We previously found that *HNMT*-1639 was associated with the allergic asthma phenotype in African American children (Anvari et al., 2015). Therefore, this SNP may have significance in disease pathophysiology and severity particularly among specific racial groups. *ABP1* 47 variant genotype was associated with decreased histamine response in our study, although this SNP has also been reported to result in a missense mutation and decreased enzyme activity which would suggest an increase in histamine accumulation and therefore increased response. However, these previous *in vitro* studies found that significantly lower enzyme activity resulting from the *ABP1* 47 SNP was only observed when the particular SNP was inherited in combination with other *ABP1* 47 SNPs. These data suggest that *ABP1* 47 variant allele containing genotypes may not be causative in reducing enzyme activity. In addition, these experiments were conducted within a relatively small number of participants with the *ABP1* 47 variant genotype (Ayuso et al., 2007). It is plausible that the *ABP1* 47 SNP may instead lead to increased activity of the enzyme. Our results indicate that more work is needed to determine the functional significance of this particular variant genotype.

We also conducted a stratified analysis among the two major racial groups included in the study (African American and Caucasian) due to our previous findings that histamine genotype was most strongly associated with allergic asthma phenotype in African American children (Anvari et al., 2015). Furthermore, the contributions of histamine to disease are particularly important to understand among African American children due to increased asthma prevalence, morbidity, and mortality in this group when compared to other racial groups as well as an increased prevalence of the allergic asthma phenotype (Akinbami, 2006). *HNMT*-1639 and *ABP1* 47 genotype were both associated with Emax among the African American children as observed in the combined cohort and results were identical in that the two SNPs were associated with increased and decreased response, respectively. *HRH4* variant genotype was also associated with an exaggerated histamine response in African American children however, the small sample size for those who possess a variant allele do not allow conclusions to be drawn regarding genotype/phenotype relationships. The functional significance of the *HRH4* 413 C/T missense mutation is unknown. With the discovery of HRH4 receptor in the 1990s and cloning of the receptor in 2000, new knowledge was gained about the effects of histamine in the body (de Esch et al., 2005). It has been revealed that histamine has far more reaching immunologic effects besides those typically associated with allergic type response at the H1 receptor. HRH4 activation not only results in production of allergic cytokines such as IL-4, IL-5, and IL-13 but also causes leukotriene production and production of pro-inflammaotry (IL-1 and IL-6) and regulatory cytokines (IL-10) (Jemima et al., 2014). Therefore, genetic variation of the HRH4 receptor is likely to have more far reaching effects than stimulating allergic response and may lead to overall alterations in the immunologic response among those with asthma and allergic disease. Attenuation of response with HRH4 inverse agonist agents, currently in clinical trials, may be even more beneficial in these patients in the treatment of asthma (Thurmond, 2015).

Previous investigations of histamine genetic variants and asthma have focused on the association between gene variants and disease. Conflicting reports exist regarding the association between SNPs such as *HNMT* 314CT and *ABP* 4107and allergic disease such as asthma, atopic dermatitis, and allergic rhinitis (Yan et al., 2000; Kennedy et al., 2008; Szczepankiewicz et al., 2010). We have previously explored histamine pathway genetic variation among children and adults with asthma. We did not observe an association between histamine genetic variation and non-phenotyped asthma. However, histamine pathway genotype was associated with the allergic asthma phenotype (Anvari et al., 2015; Raje et al., 2015). As 50–80% of those with asthma are reported to have allergic asthma, understanding the contributions of histamine and genetic variation within the histamine pathway are important. Observed differences in histamine PD response and the association with histamine pathway genetic variation among children with asthma may provide important pieces of information leading to more precise identification of asthma phenotypes that are relevant to therapeutic response. Furthermore, the use of conventional treatments, such as antihistamines, may be re-visited given newly identified functions of histamine in the body as patients with more exaggerated histamine response phenotypes and genotypes may benefit most from these types of agents.

Our study is limited by the small sample size however our results are supported by observed associations with histamine response among SNPs previously found to be associated with an allergic asthma phenotype (e.g., *HNMT*-1639 and *ABP1* 4107) suggesting that these are indeed relevant SNPs. These results require further validation in larger cohorts. We also recognize that the SNPs chosen for investigation may not indeed be causative in the observed differences in histamine response. Other variants in linkage with the investigated SNPs may be more causative or observed response may be due to combination of inherited SNPs. Our study was considerably limited in the SNPs included. Further work is needed to explore whole gene and whole genome variants that may be related to histamine response. We chose to first investigate the few SNPs that have been previously investigated and found to potentially have an association with asthma and/or allergic disease as well as variants that have a known functional significance *in vitro*. It is likely that other variants within these genes may be more important and that gene variants inherited in combination may work together in affecting histamine response. Our smaller sample size did not allow these types of analyses. It should also be considered that histamine receptors are G-protein coupled receptors and genetic variation within the downstream pathways of these receptors may also lead to differences in histamine effects and response.

In conclusion, this is the first study to translate previously observed *in vitro* effects of histamine genetic variation into apparent differences in pharmacodynamic response in humans. We identified SNPs among 3 genes responsible for histamine degradation and response that may be related to histamine pharmacodynamic effect in children with asthma. Our findings are important to further understand the contributions of histamine to asthma pathophysiology, phenotype classification, and therapeutic response profiles.

## Ethics statement

This study was carried out in accordance with recommendations of the Code of Federal Regulations, Children's Mercy Institutional Review Board, with parental permission and when appropriate child assent in accordance with the Declaration of Helsinki. This protocol was approved by the Children's Mercy Institutional Review Board.

## Author contributions

BJ was involved in overall study design, conduct of study procedures, data analysis/interpretation, and manuscript development. CS and XL was involved in study design related to modeling techniques, data analysis/interpretation, and manuscript development. HD was involved in study design related to statistical design, statistical analysis/interpretation, and manuscript development. CV was involved in study design related to genetics, conduct of study procedures related to SNP analyses, genetic data analysis/interpretation, and manuscript development.

### Conflict of interest statement

BJ received funding to conduct the study from the National Institutes of Health, National Heart Lung and Blood Institute. The other authors declare that the research was conducted in the absence of any commercial or financial relationships that could be construed as a potential conflict of interest.

## Supplementary material

Supplementary Table 1 **Investigated histamine genetic polymorphisms and known functional consequences**.

Supplementary Table 2 **Genotype comparisons of Histamine Response Pharmacodynamic Endpoints for all investigated SNPs**.
