# Supplementary material for: Genetic Variation in the Histamine Production, Response, and Degradation Pathway Is Associated with Histamine Pharmacodynamic Response in Children with Asthma
Source: Front Pharmacol. 2017 Jan 4;7:524. doi: 10.3389/fphar.2016.00524 (PMC5209333; doi:10.3389/fphar.2016.00524)
Supplement: Supplementary Table 1 — Investigated histamine genetic polymorphisms and known functional consequences. [file Table1.DOCX]

**Supplementary Table 1: Investigated histamine genetic polymorphisms and known functional consequences**

| **SNP** | **Allele change** | **Amino acid change** | **Chromosome Location** | **rs Number** | **Gene position** | **Biological Effects** | **References for variant biologic function** |
| --- | --- | --- | --- | --- | --- | --- | --- |
| *HDC* 92 | C→T | Thr31Met | 15q21-q22 | rs17740607 | Exon 2 | Not known (Missense) | Not applicable |
| *HRH1*-17 | C→T | Non coding | 3p25 | rs901865 | Exon 3, 5’UTR | Not known (5’UTR) | Not applicable |
| *HRH4* 413 | C→T | Ala138Val | 18q11.2 | rs11665084 | Exon 3 | Not known (intron variant, missense, UTR-3) | Not applicable |
| *HNMT* -1639 | C→T | Non coding | 2q22 | rs6430764 | 5’ near gene | Not known (upstream variant 2kB) | Not applicable |
| *HNMT* -464 | C→T | Non coding | 2q22 | rs2071048 | 5’ near  gene | Not known (upstream variant 2kB) | Not applicable |
| *HNMT* 314 | C→T | Thr105Ile | 2q22 | rs11558538 | Exon 4 | Altered protein structure, Decreased enzyme activity (Missense) | (24-27) |
| *HNMT* 3’UTR | A→T | Non coding | 2q22 | rs1050900 | Exon 6, 3’UTR | Not known (UTR variant 3 prime) | Not applicable |
| *ABP1* 47 | C→T | Thr16Met | 7q34-36 | rs10156191 | Exon 2 | Decreased enzyme activity (Missense) | (16, 17) |
| *ABP1* 995 | C→T | Ser332Phe | 7q34-36 | rs1049742 | Exon 2 | Decreased enzyme activity (Missense) | (16, 17) |
| *ABP1 4107* | C→G | His645Asp | 7q34-36 | rs1049793 | Exon 4 | Decreased enzyme activity (Missense) | (7) |
