# Supplementary material for: Genetic Variation in the Histamine Production, Response, and Degradation Pathway Is Associated with Histamine Pharmacodynamic Response in Children with Asthma
Source: Front Pharmacol. 2017 Jan 4;7:524. doi: 10.3389/fphar.2016.00524 (PMC5209333; doi:10.3389/fphar.2016.00524)
Supplement: Supplementary Table 2 — Genotype comparisons of Histamine Response Pharmacodynamic Endpoints for all investigated SNPs. [file Table2.DOCX]

**Supplementary Table 2:**

| SNP | Genotype | Tmax values (mean (SD)) | p value | Emax values  (mean, SD) | p value | AUEC values  (mean, SD) | p value |
| --- | --- | --- | --- | --- | --- | --- | --- |
| *HDC* 92 C/T  rs17740607 | CC | 18.7(12.1) | 0.18 | 149.5(61.3) | 0.83 | 7298.3(4201.7) | 0.86 |
|  | CT | 24.9(24.9) |  | 158.6(52.6) |  | 7853.7(4880.4) |  |
|  | TT | 14.5(3.5) |  | 131.5(18.6) |  | 6356.0(1662.3) |  |
| *HRH1* -17 C/T  rs901865 | CC | 19.2(14.7) | 0.93 | 157.1(61.3) | 0.25 | 7784.1(4651.1) | 0.41 |
|  | CT | 20.4(15.3) |  | 140.6 (53.0) |  | 6693.3(3234.7) |  |
|  | TT | 23.1(21.6) |  | 83.1(42.4) |  | 3755.0(3248.5) |  |
| *HRH4* 413 C/T  rs11665085 | CC | 20.0(15.3) | 0.64 | 149.5(57.6) | 0.61 | 7380.3(4295.4) | 0.37 |
|  | CT | 16.9(11.1) |  | 161.9(76.5) |  | 7564.6(4327.9) |  |
|  | TT | 8.27** |  | 130.2** |  | 3440.8** |  |
| *HNMT* -1639 C/T  rs 6430764 | CC | 21.3(15.4) | 0.20 | 132.6(57.7) | 0.0695  *0.02 | 6300.1(3558.4) | 0.21 |
|  | CT | 20.1(16.2) |  | 154.1(58.6) |  | 7944.4(4722.9) |  |
|  | TT | 17.5(11.6) |  | 157.8(61.6) |  | 7049.2(3710.5) |  |
| *HNMT* -464 C/T  rs2071048 | CC | 19.8(17.3) | 0.34 | 148.6(57.2) | 0.37 | 6991.4(3725.2) | 0.35 |
|  | CT | 21.1(16.2) |  | 152.0(60.7) |  | 7889.0(4775.9) |  |
|  | TT | 17.4(11.5) |  | 150.1(60.0) |  | 6725.7(3583.3) |  |
| *HNMT* 314 C/T  rs11558538 | CC | 18.8(13.0) | 0.43 | 146.1(60.7) | 0.30 | 7021.4(4160.1) | 0.21 |
|  | CT | 24.4(22.4) |  | 174.3(50.5) |  | 9232.9(4626.4) |  |
|  | TT | 14.5(10.6) |  | 153.4(0.13) |  | 7119.3(3431.0) |  |
| *HNMT* 3’UTR A/T  rs1050900 | AA | 18.9(13.6) | <0.01 | 144.6(57.6) | 0.75 | 7153.5(4558.4) | 0.18 |
|  | AT | 18.5(11.1) |  | 160.3(63.5) |  | 7388.2(3476.7) |  |
|  | TT | 40.5(36.8) |  | 156.7(48.5) |  | 10373.5(5779.4) |  |
| *ABP1* 47 C/T  rs10156191 | CC | 19.8 (16.1) | 0.87 | 167.2 59.1) | 0.11 | 8088.6(4401.0) | 0.69 |
|  | CT | 19.6(14.8) |  | 136.2(56.2) |  | 6985.0(4438.1) |  |
|  | TT | 19.4(11.3) |  | 150.5(62.0) |  | 6469.1(2855.6) |  |
| *ABP1* 995 C/T  rs1049742 | CC | 20.1(15.2) | 0.24 | 153.9(62.0) | 0.7042  *.04 | 7644.8(4555.2) | 0.33 |
|  | CT | 17.2(13.7) |  | 136.7(47.0) |  | 6105.0(2509.3) |  |
|  | TT | 32.2** |  | 134.7** |  | 8378.2** |  |
| *ABP1* 4107 C/G  rs1049793 | CC | 19.7(17.6) | 0.87 | 142.7(49.7) | 0.01  *<0.01 | 7173.5(4340.2) | 0.25 |
|  | CG | 20.4(13.3) |  | 161.4(66.8) |  | 7627.2(3954.1) |  |
|  | GG | 17.6(13.0) |  | 142.1(56.2) |  | 7149.3(5064.5) |  |

P values shown are corrected for race and asthma type (allergic vs. non-allergic)

Emax defined as maximal flux values; Tmax defined as minutes to maximal flux; AUEC defined area under the curve for flux values over time in minutes.

*Dominant genotype analysis p value

**SD not calculated due to only one participant with this genotype
